# Etiology and Risk Factors of Recurrent Urinary Tract Infections in Women in a Multidisciplinary Hospital in Romania

**DOI:** 10.3390/microorganisms13030626

**Published:** 2025-03-10

**Authors:** Corina-Ioana Anton, Ion Ștefan, Mădălina Zamfir, Constantin Florin Ghiațău, Cristian Sorin Sima, Coralia Luciana Osman, Teodora Alexia Ștefan, Adrian Streinu-Cercel

**Affiliations:** 1 Department of Infectious Diseases, “Dr. Carol Davila” Central Military Emergency University Hospital, 134 Calea Plevnei, 010242 Bucharest, Romania; corina-ioana.anton@drd.umfcd.ro (C.-I.A.); zamfir.madalinamaria@yahoo.com (M.Z.); 2 Department of Medico-Surgical and Prophylactic Disciplines, Titu Maiorescu University, 040441 Bucharest, Romania; 3 Faculty of General Medicine, Carol Davila University of Medicine and Pharmacy, 8 Eroii Sanitari Bvd, 050474 Bucharest, Romania; alexia-teodora.stefan0720@stud.umfcd.ro (T.A.Ș.); adrian.streinucercel@umfcd.ro (A.S.-C.); 4 Department of Microbiology, “Dr. Carol Davila” Central Military Emergency University Hospital, 134 Calea Plevnei, 010242 Bucharest, Romania; c_ghiatau@yahoo.com (C.F.G.); coralialuciana@gmail.com (C.L.O.); 5 Department of Infectious Diseases I, Faculty of Medicine, Carol Davila University of Medicine and Pharmacy, 020021 Bucharest, Romania; 6 National Institute for Infectious Diseases “Prof. Dr. Matei Balş”, 1 Dr. Calistrat Grozovici Street, 021105 Bucharest, Romania

**Keywords:** urinary tract infections, recurrent urinary tract infections, risk factors, bladder health

## Abstract

Background: Urinary tract infections (UTIs) are among the most prevalent bacterial infections affecting millions of people worldwide each year. They are characterized by the presence of pathogenic microorganisms in the urinary system, leading to symptoms such as frequent urination, burning sensations during urination, and lower abdominal pain. While UTIs can affect individuals of all ages and genders, they are particularly common in women due to anatomical factors. A significant concern regarding UTIs is their tendency to recur, with some patients experiencing multiple episodes within a year. Methods: This study included 674 female patients that were admitted into “Dr. Carol Davila” Central Military Emergency University Hospital in Bucharest over a period of 3 years. Results: Of the 674 women with urinary tract infection, 435 (79.3%) had more than one positive culture, and 239 (35.4%) experienced at least one recurrent urinary tract infection 6–12 months after the initial diagnosis. The mean age of women with urinary tract infection was 63 (±15.61 years). Recurrent urinary tract infections were most prevalent in those aged 65–80 years (52%). *Escherichia coli* was detected in 71% of the positive cultures. Conclusions: The high prevalence of UTIs among women is a significant public health concern that warrants attention. Women are particularly susceptible to UTIs due to various anatomical and physiological factors. This increased vulnerability underscores the importance of understanding the current epidemiological landscape of UTIs to develop effective preventive strategies.

## 1. Introduction

Urinary tract infections are a prevalent health concern among women, with an annual incidence of 10–13% and more than half of women experiencing at least one UTI in their lifetime [1].

The diagnosis and management of UTIs in primary care settings frequently rely on symptoms or a combination of symptoms and urine sample testing. However, there are increasing concerns regarding the inappropriate use of empirical antibiotics, which contribute significantly to the development of antimicrobial resistance. This underscores the importance of an accurate diagnosis to ensure appropriate antibiotic prescription and mitigate the burden of antimicrobial resistance [1,2,3].

The incidence and presentation of UTIs can vary with age, particularly in women over 65 years and those experiencing menopause. Menopause induces significant hormonal changes that can result in genitourinary syndrome of menopause, characterized by vaginal dryness and urinary symptoms, such as urgency, dysuria, and frequency [2,3]. These changes, coupled with the increasing prevalence of asymptomatic bacteriuria in older women, can lead to false-positive urine sampling results, highlighting the need for a more nuanced understanding of UTI symptoms in this population [2]. Additionally, recurrent UTI, affecting up to 3% of women, pose a particular challenge as they often necessitate repeated courses of antibiotics, further increasing the risk of antimicrobial resistance. Therefore, it is crucial to investigate whether symptoms differ in women with recurrent UTIs to ensure appropriate antibiotic treatment and minimize the development of resistance [1,2,3].

The impact of recurrent UTI extends beyond immediate health concerns, significantly affecting quality of life and presenting ongoing clinical challenges. Despite the prevalence of recurrent UTI, there is a notable lack of long-term studies to guide evaluation and treatment strategies [3,4].

Functional disability, which may limit an individual’s ability to maintain proper hygiene or empty their bladder completely, also contributes to the risk of recurrent UTI. Recent sexual intercourse is associated with an increased likelihood of bacterial introduction into the urinary tract, particularly in women, owing to their shorter urethra [2,3,4].

Additionally, a history of urogynecological surgery can alter the anatomy and function of the urinary system, potentially creating conditions conducive to recurrent infections [3,4].

To better understand recurrent UTI, it is crucial to have a comprehensive understanding of its epidemiology and characteristics. Unfortunately, there is a paucity of consistent and detailed data on recurrent UTI. Existing studies often suffer from limitations, such as a lack of microbiological confirmation, varying population demographics, and inconsistent definitions of recurrence [3,4,5]. To address this knowledge gap, we conducted a study using anonymized data and electronic hospital health records. This study aimed to provide a more accurate description of the population affected by recurrent UTI, the microbiology of these infections, and the risk factors associated with subsequent UTI episodes, potentially informing targeted and effective treatment strategies for this debilitating condition.

Recurrent UTI represents a persistent and challenging health issue, affecting millions of individuals worldwide. These infections, characterized by their frequent recurrence, significantly impact patients’ quality of life and pose a substantial burden on healthcare systems [6,7].

The female urobiome is distinguished by the presence of additional genera such as Staphylococcus as well as phyla such as Actinobacteria and Bacteroides, which are typically absent in males. These microbial communities are dynamic with fluctuations observed during periods of health and disease [8,9,10].

One striking difference between the male and female urobiomes is the prevalence of *Escherichia coli* [9,10]. This bacterium was cultured from a significantly higher proportion of healthy women (91%) than men (25%), suggesting its role as a residential organism in the female urinary tract [9].

These variations in microbial abundance and diversity are not merely descriptive; they are also associated with changes in predicted metabolic pathways, which may have implications for various urological conditions and infections [10,11]. Understanding these sex-specific differences in the urobiome is crucial for developing targeted approaches to maintain urinary tract health and to treat urological disorders in both men and women [11].

Recurrent urinary tract infections are a significant health concern, particularly in young healthy women. Despite having normal urinary tract anatomy and physiology, these women are more susceptible to vaginal colonization by uropathogens due to the increased adherence of uropathogenic coliforms to their uroepithelial cells [11,12].

Several risk factors contribute to recurrent UTI, including sexual activity, early age at first occurrence, and maternal history of UTI. Effective management approaches include continuous prophylaxis with low-dose antimicrobials, and intermittent self-treatment [11,12,13]. In postmenopausal women, mechanical and physiological factors affecting bladder emptying are the primary risk factors, and estrogen therapy has been shown to be highly effective in preventing recurrent UTI [12,13]. Ongoing research on UTI pathogenesis is expected to yield more effective and safer prevention methods for these common infections [12,13,14].

## 2. Materials and Methods

### 2.1. Study Design and Population

The retrospective cohort analysis conducted at the Department of Infectious Disease at “Dr. Carol Davila” Central Military Emergency University Hospital in Bucharest focused on patients admitted between 1 January 2021, and 31 December 2023. This study aimed to investigate recurrent UTI in a cohort of 674 patients admitted to the Department of Infectious Disease, who experienced UTI. The study population included female patients ≥ 18 years of age.

Ethical considerations were addressed through approval from the Hospital Ethics Committee (Decision No. 718/29.08.2024), and informed consent was obtained from all study participants.

### 2.2. Setting

The comprehensive electronic healthcare records used in this study served as a source of patient data, encompassing a wide range of information crucial for healthcare research and analysis. These records include detailed demographic data, providing insights into patient characteristics, such as age, sex, and socioeconomic factors. Additionally, patient charts contain extensive information on rendered healthcare services, including outpatient visits, hospitalizations, and specialized treatments.

Urinary tract infections (UTIs) were defined using a comprehensive set of criteria to ensure accurate identification and diagnosis. The first criterion was the presence of a UTI diagnosis accompanied by a prescription for specific antibiotics commonly used for treating this type of infection. Antibiotics include aminoglycosides, carbapenems, cephalosporins, fluoroquinolones, fosfomycin, nitrofurantoin, penicillins, and trimethoprim-sulfamethoxazole. This approach ensured that only cases with both clinical diagnosis and appropriate treatment were considered.

The second and third criteria focused on laboratory evidence of UTIs. A positive urine culture result, combined with either a relevant antibiotic prescription within a 3-day window of the culture date or a UTI diagnosis code within a 7-day window, was considered indicative of UTI diagnosis.

The definition of a positive urine culture was further refined based on the sample collection method. For normally sterile samples, such as those obtained through catheterization or suprapubic aspiration, a threshold of ≥1000 colony-forming units (CFU) per mL was established. For clean-catch specimens, which are more prone to contamination, a higher threshold of ≥10,000 CFU/mL was set to minimize false positive results. These criteria collectively provide a robust framework for identifying UTIs, incorporating both clinical and laboratory parameters to enhance diagnostic accuracy.

Microbiological studies in urinary tract infections (UTIs) involve a comprehensive approach to sample collection, processing, microbial identification, and antimicrobial susceptibility testing. The process involves obtaining clean-catch midstream urine, catheterized urine, or suprapubic aspiration samples, which are then cultured on selective and differential media, such as MacConkey agar, blood agar, and CLED agar. Colony counts were performed to determine significant bacteriuria, with counts exceeding 10 CFU/mL typically indicating infection.

Microbial identification was performed using biochemical tests and molecular techniques to accurately identify the causative pathogens.

### 2.3. Statistical Analysis

Statistical analysis was performed using Mann–Whitney U tests to evaluate clinical and demographic variables, as well as incidence rates. The significance threshold was set at *p* < 0.05, and all analyses were performed using SPSS software version 26.

## 3. Results

The study we conducted at the “Carol Davila” Central Military Emergency University Hospital in Bucharest provides valuable insights into the prevalence, etiology, and characteristics of UTI among female patients. Over a three-year period from January 2021 to December 2023, 674 female patients with confirmed UTIs were admitted to the Infectious Disease Department. This cohort was divided into two groups: 435 patients who experienced a single UTI episode, and 239 patients who had recurrent UTIs. The overall patient population had a mean age of 63 years (±15.61) and an average body mass index (BMI) of 30.3 (±4.3), indicating that many of the patients were overweight or obese.

Notably, this study revealed a significant age difference between patients with single and recurrent UTI episodes. Patients with recurrent UTIs were found to be considerably older, with a mean age of 73.3 years (±17.43) (Table 1).

This finding suggests that advanced age may be a risk factor for recurrent UTI in female patients. The higher prevalence of recurrent UTI in older women could be attributed to various factors, including changes in the urinary tract anatomy, decreased immune function, and increased susceptibility to infections due to comorbidities. These results underscore the importance of tailored preventive strategies and management approaches for different age groups, particularly for elderly women who may be at a higher risk of recurrent UTI.

In our study, 239 patients (35.4%) experienced recurrent UTIs, whereas 435 patients (64.5%) experienced a single episode. The prevalence of comorbidities among UTI patients was notable, with hypertension being the most common (57.4%), followed by diabetes (43.1%) and heart failure (30.4%). These findings highlight the potential relationship between chronic health conditions and susceptibility to UTI.

Several risk factors have been identified as contributing to recurrent UTI, including incomplete bladder emptying, recent urinary catheterization, urological or gynecological procedures, immobilization, and neurogenic bladder. This study also revealed the most common uropathogens responsible for these infections. *Escherichia coli* was the predominant pathogen, isolated in 40.2% of the cases, followed by *Klebsiella pneumoniae* (15.7%) and *Enterococcus faecalis* (11.4%). Notably, *E. coli* was the most prevalent organism in both single-episode and recurrent UTI cases, consistent with other studies in the field [2,15,16]. This information is crucial for developing targeted prevention strategies and treatment approaches for UTI, particularly in patients with recurrent infections or underlying health conditions.

## 4. Discussion

This study delves into the intricate and prevalent issue of UTI in medical practice. This study aimed to identify and describe the risk factors and comorbidities associated with UTIs and recurrent UTI [17,18]. The findings revealed that advanced age and various comorbidities, including diabetes, hypertension, heart failure, neurogenic bladder, and immobilization, were significantly associated with the occurrence of recurrent tract infections [18]. These results align with previous research conducted both within Romania and internationally, reinforcing the consistency of risk factors across populations and healthcare settings [18,19,20,21].

The significance of this study lies in its contribution to the existing body of knowledge on UTI and recurrent UTI, particularly in a Romanian context. By identifying specific risk factors and comorbidities, healthcare providers can better assess and manage patients at higher risk of developing recurrent UTI [18,19]. This information can be invaluable in developing targeted prevention strategies, improving patient care, and potentially reducing the burden of UTI on both patients and healthcare systems [19].

Furthermore, the consistency of findings with previous studies suggests that these risk factors may be universally applicable, allowing for more standardized approaches to UTI prevention [20,21].

Patients with recurrent urinary tract infections (UTIs) in our cohort were significantly older than those with single episodes, consistent with the existing literature that demonstrates a higher frequency of recurrent UTIs in individuals over 65 years of age. This trend is further supported by international studies, which indicate an increased prevalence of 20% among women aged ≥ 65 years [20,21,22]. Our findings revealed that recurrent UTIs were particularly prevalent among older females, affecting 32.6% of the sample population. This percentage is notably higher than that reported in previous retrospective studies, which reported that 20% of adults with recurrent UTIs were females over 65 years of age [20,21,22].

This study identified several risk factors associated with recurrent UTI, consistent with previous reports. These factors include diabetes, chronic renal failure, immobilization, heart failure, and neurogenic bladders. The higher prevalence of recurrent UTI in older females underscores the importance of targeted preventive strategies and management approaches [15,16,22].

Urinary tract infections, particularly recurrent UTI, can have a profound impact on women’s quality of life and affect both their physical and mental well-being. The symptoms of UTI, including dysuria, bladder pain, urinary urgency, and frequency, are not only physically uncomfortable but also emotionally distressing [22]. These symptoms can significantly disrupt daily activities, leading to a loss of productivity and time off work. Studies have shown that women may experience limited activity for 1–2 days per UTI episode, resulting in approximately three lost workdays annually [15,22]. Physical discomfort associated with UTI can be severe and debilitating. Dysuria, or painful urination, can make basic bodily functions a source of distress. Bladder pain can range from mild discomfort to intense stabbing sensations that interfere with movement and concentration. The constant urge to urinate coupled with the frequent need to visit the bathroom can disrupt sleep patterns, leading to fatigue and decreased overall well-being [15]. In some cases, UTI can progress to more serious conditions, such as kidney infections, if left untreated, further compromising health and quality of life [15,16].

The impact of UTI extends beyond immediate physical symptoms. Women with recurrent UTI often find themselves in a constant state of vigilance and are always aware of the potential onset of another infection [15,16,22]. This hyperawareness can lead to changes in behavior such as excessive water consumption, frequent bathroom visits, and avoidance of certain activities or situations that they perceive as potential triggers for UTI [15,16]. Such lifestyle modifications, although sometimes necessary, can be restrictive and may contribute to a sense of loss of control over one’s life. The psychological toll of recurrent UTI in women was equally significant [2,16]. The unpredictable and painful nature of UTI episodes can induce anxiety and depression in up to 62% of female patients, with mental health scores below average in as many as 81% of women affected [2,16]. The high prevalence of mental health issues among women with recurrent UTI is a clear indication of the condition’s far-reaching psychological impact [2,15,16]. Constant worry about when the next infection might occur can lead to chronic stress, which, in turn, can weaken the immune system, potentially making women more susceptible to future infections and creating a vicious cycle [16].

Furthermore, recurrent UTI can strain personal relationships and intimacy. Approximately 34% of women with recurrent UTI report a negative impact on their physical sexual intimacy, while 57% believe that the chronic and recurrent nature of the condition adversely affects other social relationships [15,16]. The impact on sexual intimacy is particularly significant as UTI can cause pain or discomfort during sexual intercourse, leading to avoidance of sexual activity [16]. This can create tension and misunderstanding in romantic relationships, potentially leading to feelings of guilt, inadequacy, or resentment [2,16].

The social implications of recurrent UTI extend beyond intimate relationships. Women may find themselves canceling social engagements, limiting travel, or avoiding certain activities because of the unpredictability of their symptoms [2,15,16]. This can lead to social isolation and a decreased sense of participation in enjoyable activities. Additionally, the stigma associated with UTI and the discomfort of discussing such personal health issues can make it challenging for women to seek support from friends and family, further exacerbating their feelings of isolation [2,16].

The economic impact of recurrent UTI is noteworthy. Beyond lost workdays, significant healthcare costs are associated with frequent doctor visits, diagnostic tests, and medications. For some women, the cost of managing recurrent UTI can be a substantial financial burden, particularly if they require specialized treatment or if their condition leads to complications requiring hospitalization [2,16,23].

Healthcare providers need to address not only the physical symptoms, but also the psychological and social aspects of the condition. This may include providing mental health support, offering guidance on managing the impact of the condition on relationships, and exploring both medical and lifestyle interventions to reduce the frequency of infections [2,23].

Repeated infections with the same uropathogen are a hallmark of recurrent tract infections, as observed across all bacterial species identified through culture methods [2]. This pattern of recurrence suggests that certain uropathogens possess mechanisms that persist within the urinary tract or recolonize it after the initial treatment. However, the frequency of such recurrences varies depending on the uropathogen involved [2,16].

This variation may be attributed to differences in virulence factors, antibiotic resistance profiles, or the ability of certain bacteria to form biofilms or invade bladder epithelial cells, thereby evading host immune responses and antibiotic treatment [2,23].

The lack of documented patient education on preventing recurrent urinary tract infections highlights a potential gap in comprehensive care for individuals with this condition [2,16].

Proper education on preventive measures, such as maintaining adequate hydration, practicing good hygiene, and adhering to prescribed treatment regimens, could significantly reduce the incidence of recurrent UTI [2,23]. This absence of documented education suggests a need for healthcare providers to implement and record structured patient education programs to empower patients to manage their condition effectively [23]. The referral patterns for gynecological evaluation in patients with recurrent UTI provide insights into the demographic distribution and management approach for this condition [2,23].

Patient education about proper hygiene practices, dietary modifications, and early recognition of symptoms can empower women to play a more proactive role in managing their condition [23]. The impact of recurrent UTI on women’s quality of life is complex and far-reaching, affecting their physical health, mental well-being, social relationships, and economic stability [3,23]. A comprehensive approach to managing this condition is essential to alleviate the burden on affected women and to improve their overall quality of life. By addressing both the medical and psychosocial aspects of recurrent UTI, healthcare providers can help women regain control over their health and well-being, ultimately leading to improved outcomes and better quality of life [3].

The impact of recurrent urinary tract infections on sexual function remains a critically understudied area within an already-neglected field of research. This gap in knowledge is particularly concerning, given the significant role that sexual activity plays in UTI occurrence and recurrence [3].

The established link between sexual intercourse and UTI has led many women to develop complex emotional responses to sex, including feelings of disgust and fear. These negative associations can have far-reaching consequences, potentially disrupting intimate relationships and the overall quality of life [3,23].

Despite the lack of formal research, the importance of this topic is evident in patient-driven discussions in online support forums. Qualitative analyses of these forums have revealed that sex is a major concern for women with recurrent UTI [3,24].

The psychological impact of associating sexual activity with painful infections can be profound, leading to anxiety, decreased libido, and avoidance of intimate contacts [6,24]. Furthermore, the strain placed on relationships due to these issues can be substantial, affecting not only individuals with recurrent UTI but also their partners [6,25]. This disconnect between the lived experiences of patients and the focus of medical research highlights the urgent need for more comprehensive studies on the sexual and psychosocial implications of recurrent UTI [25,26].

## 5. Conclusions

Urinary tract infection and recurrent UTI are common problems in outpatient’s visits. About one-third of patients with recurrent UTI are male. Urology and gynecological referral were infrequently requested as part of the evaluation process for patients with recurrent UTI. There was a lack of use of other interventions such as topical estrogen in postmenopausal women.

The overall process of caring for patients with recurrent UTI lacks adequate documentation and focus on individuals’ preference rather than an organized systematic approach. This highlights the need for improved standardization in the evaluation, treatment, and follow-up of patients with recurrent UTIs to ensure optimal outcomes and reduce the burden of this condition on both patients and healthcare systems.

Limitations: It is important to note the limitations of our study, primarily its single-center retrospective nature, which may introduce selection bias. Future multicenter, prospective studies could provide more comprehensive insights into the prevalence and risk factors of recurrent UTI across diverse populations.

## Figures and Tables

**Table 1:** Characteristics of patients with UTI and recurrent UTI.

Parameter	Total UTI n (%) N = 674	Single UTI n (%) N = 435	Recurrent UTI n (%) N = 239	*p*-Value
**Age**	63 ± 15.61	57.5 ± 16.46	73.3 ± 17.43	<0.002
20–30	90 (13.3)	61(14.1)	29 (12.1)	<0.003
31–40	125 (18.5)	107 (24.6)	18 (7.5)	<0.001
41–50	103 (15.2)	72 (16.5)	31(12.9)	<0.003
51–60	186 (27.5)	105 (24.1)	81 (33.9)	<0.002
≥65	130 (19.2)	52 (12)	78 (32.6)	<0.002
BMI (Mean ± SD)	30.3 ± 4.3	28.1 ± 5.2	29.6 ± 4.9	0.341
**Comorbidities**				
Chronic renal disease	174 (25.9)	43 (9.8)	131 (54.8)	<0.001
Hypertension	387 (57.4)	241 (55.4)	146 (61.1)	0.002
Malignancies	87 (12.9)	23 (5.2)	64 (26.7)	0.102
Heart failure	205 (30.4)	109 (25.1)	96 (40.1)	0.82
Stroke	214 (31.7)	121 (27.8)	93 (38.9)	0.210
Diabetes	291 (43.1)	112 (25.7)	179 (74.9)	<0.002
**Risk factors**				
Recent urinary catheter	318 (47.1)	117 (26.8)	201 (84.1)	<0.002
Neurogenic bladder	23 (3.4)	2 (0.4)	21 (8.7)	<0.003
Polycystic kidney disease	2 (0.2)	1 (0.2)	1 (0.4)	0.106
Urological and gynecological procedures	31 (4.6)	3 (0.6)	28 (11.7)	<0.001
Immobilization	92 (13.6)	7 (1.6)	85 (35.5)	<0.002
Incomplete bladder emptying	10 (1.4)	1 (0.2)	9 (3.7)	
**Etiologic agent**				
*Escherichia coli*	271 (40.2)	127 (29.1)	144 (60.2)	0.86
*Klebsiella pneumonia*	106 (15.7)	49 (11.2)	57 (23.8)	0.91
*Enterococcus faecalis*	77 (11.4)	41 (9.4)	36 (15.1)	<0.001
*Pseudomonas aeruginosa*	52 (7.7)	34 (7.8)	18 (7.5)	<0.002
*Streptococcus agalactiae*	47 (6.9)	36 (8.2)	11 (4.6)	<0.002
*Staphilococcus aureus*	62 (9.1)	42 (9.6)	20 (8.3)	<0.001
*Candida auris*	59 (8.7)	28 (6.4)	31 (13.1)	<0.001

## Data Availability

The data presented in this study are available on request from the corresponding author due to legal reason.
